# Real-time detection of laryngopharyngeal cancer using an artificial intelligence-assisted system with multimodal data

**DOI:** 10.1186/s12967-023-04572-y

**Published:** 2023-10-07

**Authors:** Yun Li, Wenxin Gu, Huijun Yue, Guoqing Lei, Wenbin Guo, Yihui Wen, Haocheng Tang, Xin Luo, Wenjuan Tu, Jin Ye, Ruomei Hong, Qian Cai, Qingyu Gu, Tianrun Liu, Beiping Miao, Ruxin Wang, Jiangtao Ren, Wenbin Lei

**Affiliations:** 1 Otorhinolaryngology Hospital, The First Affiliated Hospital, Sun Yat-Sen University, Guangzhou, 510080 Guangdong China; 2 School of Computer Science and Engineering, Guangdong Province Key Lab of Computational Science, Sun Yat-Sen University, Guangzhou, 510006 Guangdong China; 3 Department of Otolaryngology-Head and Neck Surgery, Nanfang Hospital, Southern Medical University, Guangzhou, Guangdong China; 4 Department of Otolaryngology-Head and Neck Surgery, The Third Affiliated Hospital of Sun Yat-Sen University, Guangzhou, Guangdong China; 5 Department of Otolaryngology-Head and Neck, Sun Yat-Sen Memorial Hospital, Sun Yat-Sen University, Guangzhou, Guangdong China; 6 Department of Otorhinolaryngology-Head and Neck Surgery, The Sixth Affiliated Hospital of Sun Yat-Sen University, Guangzhou, Guangdong China; 7 Department of Otolaryngology-Head and Neck Surgery, Shenzhen Secondary Hospital and First Affiliated Hospital of Shenzhen University, Shenzhen, Guangdong China; 8 Shenzhen Institute of Advanced Technology, Chinese Academy of Sciences, Shenzhen, Guangdong China

**Keywords:** Head and neck tumour, Deep-learning models, Diagnostic, Laryngoscopic, Multicentre, Real-time

## Abstract

**Background:**

Laryngopharyngeal cancer (LPC) includes laryngeal and hypopharyngeal cancer, whose early diagnosis can significantly improve the prognosis and quality of life of patients. Pathological biopsy of suspicious cancerous tissue under the guidance of laryngoscopy is the gold standard for diagnosing LPC. However, this subjective examination largely depends on the skills and experience of laryngologists, which increases the possibility of missed diagnoses and repeated unnecessary biopsies. We aimed to develop and validate a deep convolutional neural network-based Laryngopharyngeal Artificial Intelligence Diagnostic System (LPAIDS) for real-time automatically identifying LPC in both laryngoscopy white-light imaging (WLI) and narrow-band imaging (NBI) images to improve the diagnostic accuracy of LPC by reducing diagnostic variation among on-expert laryngologists.

**Methods:**

All 31,543 laryngoscopic images from 2382 patients were categorised into training, verification, and test sets to develop, validate, and internal test LPAIDS. Another 25,063 images from five other hospitals were used as external tests. Overall, 551 videos were used to evaluate the real-time performance of the system, and 200 randomly selected videos were used to compare the diagnostic performance of the LPAIDS with that of laryngologists. Two deep-learning models using either WLI (model W) or NBI (model N) images were constructed to compare with LPAIDS.

**Results:**

LPAIDS had a higher diagnostic performance than models W and N, with accuracies of 0·956 and 0·949 in the internal image and video tests, respectively. The robustness and stability of LPAIDS were validated in external sets with the area under the receiver operating characteristic curve values of 0·965–0·987. In the laryngologist-machine competition, LPAIDS achieved an accuracy of 0·940, which was comparable to expert laryngologists and outperformed other laryngologists with varying qualifications.

**Conclusions:**

LPAIDS provided high accuracy and stability in detecting LPC in real-time, which showed great potential for using LPAIDS to improve the diagnostic accuracy of LPC by reducing diagnostic variation among on-expert laryngologists.

**Supplementary Information:**

The online version contains supplementary material available at 10.1186/s12967-023-04572-y.

## Background

Laryngopharyngeal cancer (LPC), including laryngeal cancer (LCA) and hypopharyngeal cancer, is the second most common malignancy among head and neck tumours, with more than 130,000 deaths reported in 2020 [1]. Laryngoscopy biopsy is the gold standard for diagnosing LPC [2, 3]. In-office transnasal flexible electronic endoscopy can intuitively examine the laryngopharynx, making it the most effective device for detecting LPC [4, 5]. The limited resolution and contrast of white light can lead to the neglect or missed diagnosis of superficial mucosal cancers, even by experienced endoscopists [6, 7]. This can lead to patients being diagnosed at a later stage and thus having to undergo a multimodal treatment approach, resulting in poor prognosis and reduced quality of life [8–10]. Furthermore, a precautionary biopsy is usually prescribed to avoid the missed diagnosis of early-stage cancer, resulting in overtreatment and emotional stress to patients [11]. Recently, endoscopic systems with narrow-band imaging (NBI), which can improve the clarity and identification of epithelial and subepithelial microvessels, have played a critical role in the early diagnosis of LPC with high specificity and sensitivity [12–14]. However, owing to the relatively long professional training and accumulation of clinical experience, this technology is at high risk of missing suspicious LPCs in endoscopy examinations in hospitals with inexperienced laryngologists, underdeveloped regions, and countries with large numbers of patients [15, 16].

Recently, artificial intelligence (AI) has shown great potential in assisting doctors in various medical fields with their diagnoses [17–19]. Particularly, deep learning techniques based on deep convolutional neural networks (DCNN) have demonstrated extraordinary capabilities for medical image classification, detection, and segmentation [20, 21]. Benefiting from its super-resolution performance on microscopic images, AI can automatically infer complex microscopic imaging structures (i.e., abnormalities in the extent and colour intensity of mucosal tubular branches) and identify quantitative pixel-level features [22], which are usually indistinguishable from the human eye. Several studies have demonstrated the feasibility and effectiveness of deep learning for lesion detection and the pathological classification of endoscopic images. Unfortunately, there are still several limitations to the existing research, particularly concerning laryngoscopy. Despite the real-time nature of endoscopy, current research is limited to detecting a single image [23, 24], and there is a lack of studies integrating AI into dynamic videos. Additionally, most existing studies focus on a single light source, including the application of white-light imaging (WLI) and NBI images [25–27], without considering the fusion of their multimodal features, which may increase the possibility of missed diagnosis and misdiagnosis.

We developed a DCNN-based Laryngopharyngeal Artificial Intelligence Diagnostic System (LPAIDS) that incorporates NBI and WLI multimodal features for endoscopic diagnosis of laryngopharyngeal carcinoma. We aimed to investigate whether the model can achieve expert-comparable performance and be applied in real-world laryngoscopy scenarios. Therefore, to fully simulate the clinical scene of endoscopy in the real world, we extracted the video frames of laryngoscopy videos during real-world endoscopy for model training. The diagnostic performance was validated using a time-series test set and external test sets from five other hospitals, and its real-time detection performance was verified using video. Additionally, we compared the implementation of this LPAIDS with that of laryngologists of different qualifications using endoscopist-machine competition.

## Methods

### Study design and participants

This retrospective, multicentre diagnostic study was conducted in six tertiary hospitals in China. We retrospectively obtained a video of the electronic laryngoscope at the First Affiliated Hospital of Sun Yat-sen University (FAHSYSU). We extracted the required video frames, including NBI and WLI images, for the development, validation, and internal testing of the LPAIDS. Time-series sets were used to train, validate, and test the model to better evaluate the practicability in clinical practice.

To generalise the applicability of the LPAIDS, laryngoscopic images of patients were collected from the following five hospitals in China for an external test: Sun Yat-sen Memorial Hospital of Sun Yat-sen University (SYMSYSU), Nanfang Hospital of Southern Medical University (NHSMU), First Affiliated Hospital of Shenzhen University (FAHSU), Third Affiliated Hospital of Sun Yat-sen University (TAHSYSU), and Sixth Affiliated Hospital of Sun Yat-sen University (SAHSYSU). To evaluate the efficacy of LPAIDS in real time, videos stored in FAHSYSU from 1 December 2021 to 31 March 2022 were collected for performance testing, and 200 videos were randomly selected for performance comparison with different levels of endoscopists.

Enrolled laryngoscopic images or videos were obtained from consecutive patients aged ≥ 18 years who underwent laryngoscopy. According to the World Health Organization classification of tumours, the pathological diagnosis was confirmed by two board-certified pathologists using haematoxylin–eosin-stained tissue slides, which served as the gold standard for judgment. The exclusion criteria were patients who had previously undergone laryngeal surgery or chemotherapy and radiotherapy for LPC and those without a histologically confirmed pathological diagnosis. Patients with laryngopharyngeal lesions (including carcinomas of the larynx and hypopharynx) with histologically proven malignancies were eligible for this study. For normal controls or participants with histologically confirmed benign neoplasms (such as vocal cord polyps, vocal nodules, and vocal cord leucoplakia), no specific exclusion criteria were available regarding clinical characteristics or demographics.

### Laryngoscopy and image quality control

All laryngoscopies in this study were performed in daily clinical practice as screening or pretreatment examinations. The equipment used in this study included different models of standard laryngoscopy (ENF-VT2, ENF-VH, ENF-VT3, ENF-V2, or ENF-V3; Olympus Medical Systems, Tokyo, Japan; EV-N, EV-NC20, or EV-NE; Xion, Berlin, Germany) and video systems (VISERA ELITE OTV-S190, EVIS EXERA III CV-190, EVIS LUCERA CV-260SL, and VISERA Pro OTV-S7Pro; Olympus Medical Systems, Tokyo, Japan; XN HD3, Xion, Berlin, Germany). All laryngoscopy videos were stored in AVI or MP4 format, and images were stored in JPG format at the six hospitals.

Laryngoscopy video frames were extracted from the three doctoral students. The extracted video frames contained different representative positions and angles of the laryngopharynx and covered various activities of the laryngopharynx. Each patient captured no more than 10 video frames and avoided repeated sampling at the same location. Nasopharyngeal, oropharyngeal, and images of lesions that were difficult to assess because of poor visual field quality due to active bleeding, thick buffy coat, mucus, halos, defocus, blurring, and reflections were removed. Three highly experienced laryngoscopists at FAHSYSU, each with at least 5 years of experience in laryngoscopy and conducting more than 3000 laryngoscopy examinations, carefully reviewed all images and selected representative LPC and non-cancer images according to the pathologic reports. Three laryngoscopists independently delineated all cancer lesions to outline the boundaries of the actual lesion area within the images. Image annotation used the tool labelme (https://github.com/wkentaro/labelme). Annotated images were used as mask layers for model training. All images were reviewed using crosschecking and expert reviews for quality control to avoid individual bias. Annotations and delineations in the images were only finalised when a consensus was reached between at least two endoscopists. When two endoscopists could not agree, a senior laryngeal specialist with at least 20 years of experience in laryngopharyngeal tumours made the final decision.

### Dataset distribution

**Fig. 1:**
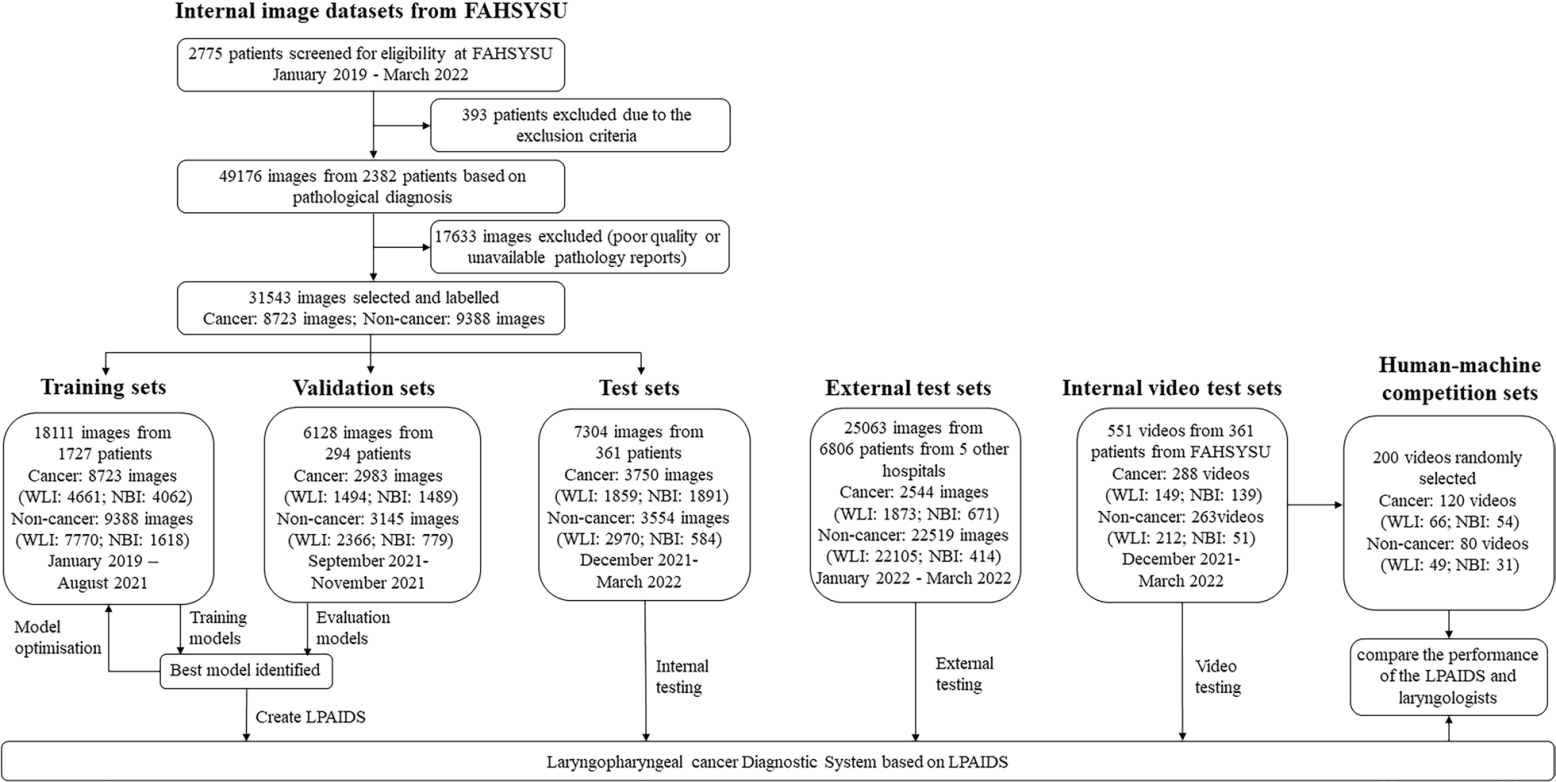
Flowchart for development and evaluation of the LPAIDS for laryngopharyngeal cancer diagnosis. LPAIDS: Laryngopharyngeal Artificial Intelligence Diagnostic System

The dataset distribution of this study is shown in Fig. 1. Laryngoscopy videos of 2775 patients were retrospectively obtained from the database of the Laryngoscopy Center of FAHSYSU, and 393 patients were excluded based on the exclusion criteria. Overall, 49,176 laryngoscopy video frames were extracted from the remaining 2382 patients. After quality assessment, 17,633 frames were discarded because of poor quality or unavailable pathology reports. For patients with cancer, only images of cancerous lesions were included. Images of normal controls and benign lesions were included for patients without cancer. The remaining 31,543 images were used for model training, temporal verification, and temporal testing, and 1005 videos were used for model temporal verification and temporal testing. A dataset of 25,293 images of 6806 patients from five other centres was considered as an external test set. The patients were independent in the different datasets. Additionally, a human–machine competition set of 200 videos randomly selected from the temporal internal video test sets was used to compare the performance of LPAIDS and laryngologists with different qualifications. All videos or images were anonymised before recording to protect the patients’ privacy.

### Development of models

**Fig. 2:**
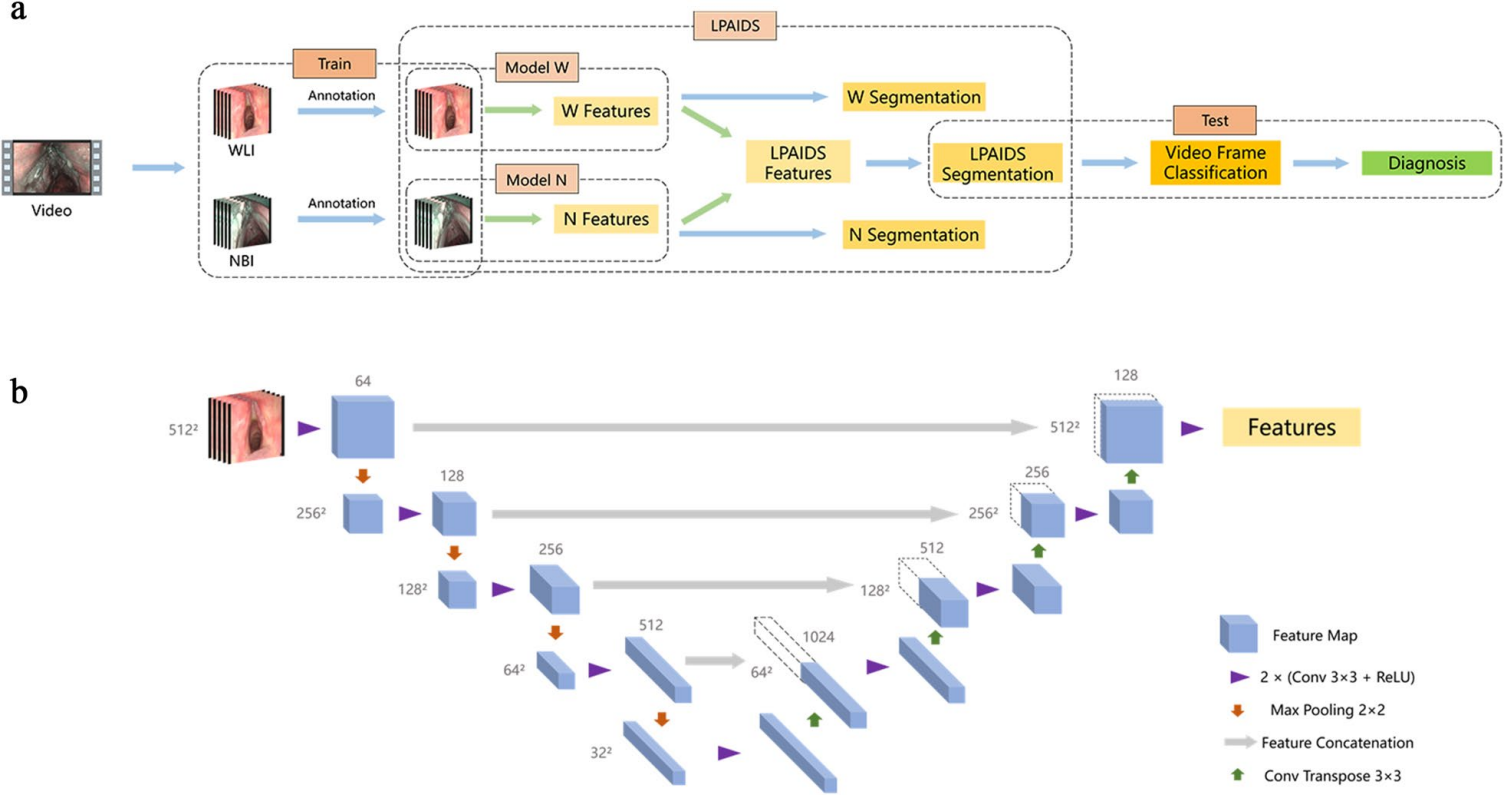
Workflow and architecture of LPAIDS. **a** Procedure for detecting LPC from laryngoscopy videos. The WLI and NBI laryngoscopy video frames were extracted from laryngoscopy videos. After screening and annotation by highly experienced laryngoscopists, the images were fed into the model to localize the area with possible tumours; the diagnoses were based on the shape and size of the tumour area. Three pre-trained convolutional neural network models (model W, model N, and LPAIDS, based on U-Net) were developed to obtain the feature vectors from the WLI, NBI, and all images, respectively. **b** The detailed neural network architecture of LPAIDS based on U-Net. LPAIDS: Laryngopharyngeal Artificial Intelligence Diagnostic System; LPC: laryngopharyngeal cancer; NBI: narrow-band imaging; WLI: white-light imaging

Since the diagnosis was a classification task, we conducted a diagnosis based on the output of semantic segmentation models. As shown in Fig. 2, first, the semantic segmentation models were used to predict tumour regions on each video frame. Second, we decided whether the video frames were classified as cancer according to the size and shape of the regions. Finally, we conducted a diagnosis based on the continuous LPC regions in the video frame sequence. The model’s algorithm was based on the concept of U-Net [28], which consists of an encoder and decoder to extract and combine different levels of features. The encoder included four convolutional blocks with two 3 × 3 layers, each followed by a rectified linear unit (ReLU) and a 2 × 2 max pooling operation with a stride of 2 for downsampling. The decoder comprised four upsampling blocks with a concatenation of the current feature map and the feature map correspondingly cropped from the encoder, each followed by two 3 × 3 convolutional layers and a ReLU. Finally, a 1 × 1 convolutional layer was used to map the feature vectors of each pixel in all channels to the predicted classes. To compare the performance of WLI images solely, NBI images solely, and WLI combined with NBI images in diagnosing LPC, model W (training and testing with WLI images), model N (training and testing with NBI images), and LPAIDS (training and testing with all images) were developed. The images in the training and testing sets were resized to 512 × 512 pixels before being fed into the models. We used several data augmentation strategies to improve the feature extraction capability of the models, including horizontal flips, rotations, colour jitters, blurs, and noise.

### Testing of the models in still images

First, we tested the performance of LPAIDS in identifying LPC in patients using the independent temporal image test sets from FAHSYSU. Furthermore, we used WLI and NBI images in the internal temporal image test sets. We compared the diagnostic performance of LPAIDS and model W in WLI images and that of LPAIDS and model N in NBI images. Subsequently, we assessed the robustness of LPAIDS using five external test sets from SYMSYSU, NHSMU, FAHSU, TAHSYSU, and SAHSYSU, each with a small number of patients with LPC.

### Testing of the models in the temporal video datasets and comparison with laryngologists

We used clip videos as the test sets to assess the applicability of LPAIDS in the clinic. With the guidance of a laryngeal expert, three doctoral students de-identified and clipped the videos. The length of the video clips was 8–25 s per lesion. Similarly, we used WLI and NBI videos in the temporal internal video test sets. We compared the diagnostic performance of LPAIDS and model W in WLI videos and the diagnostic performance of LPAIDS and model N in NBI videos.

For further performance evaluation of the LPAIDS, we randomly selected 200 videos (including 115 WLI and 85 NBI videos) from the temporal video test sets. Subsequently, we mixed them in a scrambled order and de-identified them. Ten laryngologists with varying degrees of expertise (expert, senior, resident, and trainee) were asked to complete 200 test videos independently, and the results were compared with those of the LPAIDS. The 10 laryngologists were involved in selecting and annotating all datasets and were blinded to the demographics and final histopathologic results of patients on the test sets. The expert laryngologist was a professor with > 20 years of experience in endoscopic procedures. The three senior laryngologists were attending doctors with more than 5 years of experience who had completed clinical and specific endoscopic training. The three laryngologist residents had more than 3 years of endoscopic experience. The three trainees were interns with 1 year of endoscopic experience.

### Outcomes

The primary outcomes were the diagnostic accuracy, sensitivity, specificity, positive predictive value (PPV), and negative predictive value (NPV) of the models for identifying cancerous lesions. Accuracy was defined as the percentage of correctly classified individuals among all participants. Sensitivity and specificity were determined using the percentage of pathologically confirmed cancerous cases and that of negative controls, respectively. PPV was used to indicate the ratio of correctly predicted positive samples to all positive samples, and NPV was used to indicate the ratio of correctly predicted negative samples to all negative samples. To visually interpret the learned model, we used a heat map overlaid on the input image to examine the testing images to determine whether the salient regions in the saliency map corresponded to the region in interest for decision-making. The segmentation predictions of images comprised predicted values at each pixel, which indicated it was cancer or background. The high values led to cancer, and the low values led to the background. We assigned different colours to pixels based on the predicted values to obtain the heatmap. Additionally, we used intersection-over-union (IOU) to measure the image segmentation performance of the model. The IOU was based on the annotations and predictions of the LPC regions.

### Statistical analysis

The thresholds for the final decision generated were based on the statistical data. First, we applied dilation and erosion operations to eliminate voids and noises to filter the shape of the tumour regions, which were always connected. For still images, we chose the classification threshold from 8 × 8, 16 × 16, 32 × 32, 64 × 64, 128 × 128, and 256 × 256, where 8 × 8 and 256 × 256 led to the minimum and maximum values, respectively. Threshold 32 × 32 had the best performance. Therefore, we determined that images with areas of the regions exceeding 32 × 32 pixels were classified as cancer. For videos, we selected the diagnosis threshold from different time points, including 0.5 s, 1 s, 1.5 s, 2 s, 2.5 s, and 3 s, where 0.5 s and 3 s led to the minimum and maximum values, respectively. Threshold 2 s had the best performance. Therefore, we determined that the videos with consecutive cancerous frames exceeding 2 s were diagnosed as cancer.

To assess the performance of the LPAIDS and laryngologists in identifying cancerous lesions, metrics, including accuracy, sensitivity, specificity, PPV, and NPV, were evaluated by calculating the 95% confidence interval (CI) using the Clopper–Pearson method. Performance comparison between LPAIDS and laryngologists using two-sided McNemar test. The receiver operating characteristic (ROC) curve, which was created according to the true positive rate (sensitivity) and false positive rate (1 – specificity), was employed to show the diagnostic ability of the models in discriminating patients with LPC from controls. The area under the ROC curve (AUC) value was calculated. Larger AUC values indicated better diagnostic performance. Inter-observer and intra-observer agreements of the LPAIDS and laryngologists were computed using Cohen’s kappa coefficient. Statistical significance was set at *p* < 0·05. Statistical analyses were performed using SPSS (version 22.0; IBM, USA) or Python (version 3.7.13).

## Results

### Baseline characteristics

Overall, 2382 individuals from FAHSYSU and 6806 individuals from five other hospitals were enrolled in this study. The baseline patient characteristics are shown in Table 1. The prevalence of LPC was 32·3% (558 of the 1727 patients) in the training sets, 41·2% (121 of the 294 patients) in the intrinsic verification sets, 41·3% (149 of 361 the patients) in the internal test sets, 4·2% (94 of the 2242 patients) in the SYMSYSU external test sets, 3·7% (68 of the 1852 patients) in the NHSMU, 2·6% (39 of the 1491 patients) in the FAHSU, 8·0% (42 of the 528 patients) in the TAHSYSU, and 6·9% (48 of the 693 patients) in the SAHSYSU.

**Table 1:** Baseline characteristics

Characteristics	FAHSYSU validation (n = 2382)			External validation (n = 6806)				
	Training (n = 1727)	Verification (n = 294)	Testing (n = 361)	SYMSYSU (n = 2242)	NHSMU (n = 1852)	FAHSU (n = 1491)	TAHSYSU (n = 528)	SAHSYSU (n = 693)
Age(years), mean(range)	51·0 (18–93)	52·9 (20–86)	53·7 (19–86)	44·8 (18–90)	42·4 (18–88)	44·6 (18–78)	43·7 (18–81)	44·2 (18–86)
Sex								
Male	1158 (67·1%)	216 (73·5%)	273 (75·6%)	1181 (52·7%)	1008 (54·4%)	741 (49·7%)	282 (53·4%)	345 (49·8%)
Female	569 (32·9%)	78 (26·5%)	88 (24·4%)	1061 (47·3%)	844 (45·6%)	750 (50·3%)	246 (43·6%)	348 (50·2%)
Laryngeal cancer	453 (26·2%)	97 (33·0%)	121 (33·5%)	73 (3·3%)	57 (3·1%)	32 (2·1%)	34 (6·4%)	39 (5·7%)
Hypopharyngeal cancer	105 (6·1%)	24 (8·2%)	28 (7·8%)	21 (0·9%)	11 (0·6%)	7 (0.5%)	8 (1·5%)	9 (1·3%)
Benign disease	443 (25·7%)	49 (16·6%)	63 (17·4%)	178 (7·9%)	116 (6·3%)	63 (4·2%)	48 (9·1%)	57 (8·2%)
No disease	726 (42·0%)	124 (42·2%)	149 (41·3%)	1970 (87·9%)	1668 (90·0%)	1389 (93·2%)	438 (83·0%)	588 (84·8%)

FAHSU: First Affiliated Hospital of Shenzhen University; FAHSYSU: First Affiliated Hospital of Sun Yat-sen University; NHSMU: Nanfang Hospital of Southern Medical University; TAHSYSU: Third Affiliated Hospital of Sun Yat-sen University; SAHSYSU: Sixth Affiliated Hospital of Sun Yat-sen University; SYMSYSU: Sun Yat-sen Memorial Hospital of Sun Yat-sen University

### Performance of the LPAIDS in internal sets

**Fig. 3:**
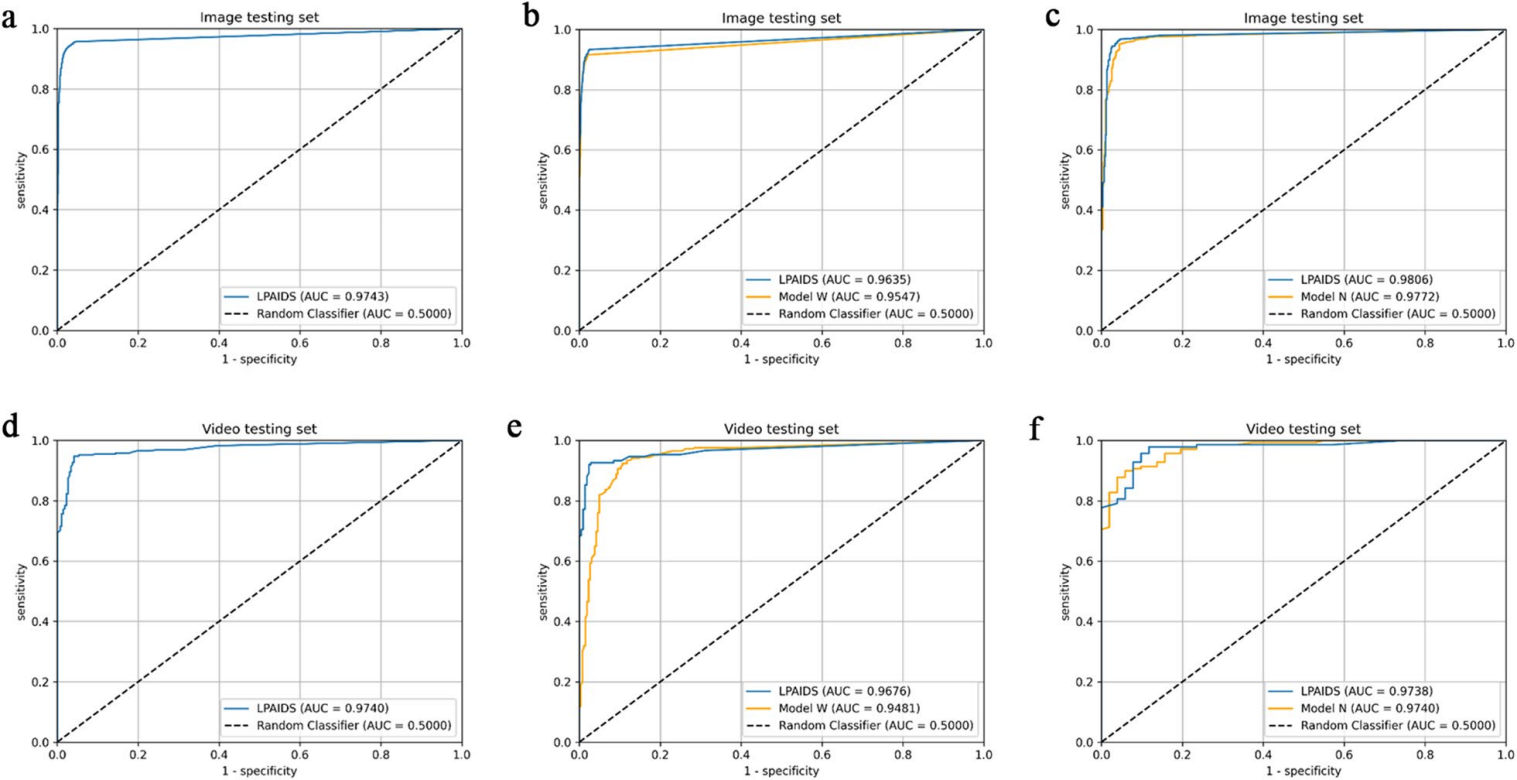
Performance of LPAIDS for identifying laryngopharyngeal cancer in the internal image and video datasets. **a** ROC curves of LPAIDS using all images in the internal image test set. **b** ROC curves of LPAIDS and model W using WLI images in the internal image test set. **c** ROC curves of LPAIDS and model N using NBI images in the internal image testing set. **d** ROC curves of LPAIDS using all videos in the internal video test sets. **e** ROC curves of LPAIDS and model W using WLI videos in the internal video test sets. **f** ROC curves of LPAIDS and model N using NBI videos in the internal video test sets. LPAIDS: Laryngopharyngeal Artificial Intelligence Diagnostic System; ROC: receiver operating characteristic; NBI: narrow-band imaging; WLI: white-light imaging

**Fig. 4:**
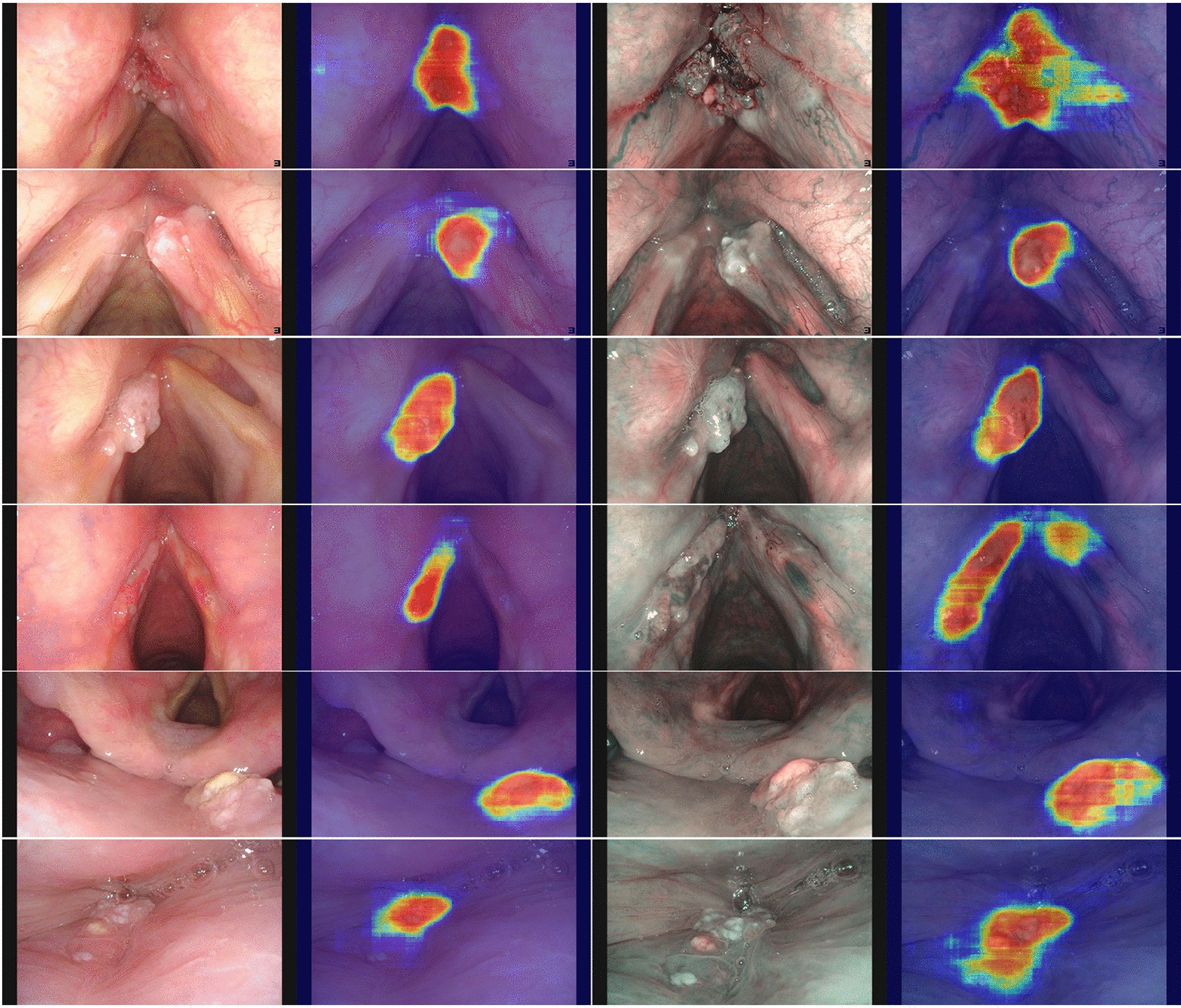
Representative attention maps obtained by LPAIDS for identifying laryngopharyngeal cancer. The attention map is shown as a heatmap superimposed on the original image, where warmer colors indicate higher saliency. LPAIDS: Laryngopharyngeal Artificial Intelligence Diagnostic System

As shown in Table 2, in the image test sets, the diagnostic accuracy of the LPAIDS tested in all images was 0·956 (95% CI 0·951–0·960), with sensitivity, specificity, PPV, and NPV of 0·948 (95% CI 0·941–0·955), 0·964 (95% CI 0·958–0·970), 0·965 (95% CI 0·959–0·971), and 0·946 (95% CI 0·939–0·953), respectively. Compared with models using single modality imaging, the LPAIDS tested in WLI images had higher accuracy (0·957 vs 0·948), sensitivity (0·918 vs 0·885), NPV (0·950 vs 0·932), and comparable specificity (0·980 vs 0·988) and PPV (0·967 vs 0·978) compared with model W. Similarly, the LPAIDS tested in NBI images had higher accuracy (0·954 vs 0·935), specificity (0·878 vs 0·798), PPV (0·963 vs 0·940), NPV (0·923 vs 0·917), and comparable sensitivity (0·977 vs 0·978) compared with model N. In the video test sets, the diagnostic accuracy, sensitivity, specificity, PPV, and NPV of the LPAIDS tested for all videos were 0·949 (95% CI 0·931–0·968), 0·948 (95% CI 0·922–0·974), 0·951 (95% CI 0·924–0·977), 0·955 (95% CI 0·930–0·979), and 0·943 (95% CI 0·916–0·971), respectively. Compared with models using single modality imaging, the LPAIDS tested in WLI videos had higher accuracy (0·950 vs 0·945), sensitivity (0·919 vs 0·886), NPV (0·945 vs 0·925), and comparable specificity (0·972 vs 0·986) and PPV (0·958 vs 0·978) compared with model W. Similarly, the LPAIDS tested in NBI videos had higher accuracy (0·947 vs 0·926), sensitivity (0·978 vs 0·971), specificity (0·863 vs 0·804), PPV (0·951 vs 0·931), and NPV (0·936 vs 0·911) compared with model N. The representative videos of LPAIDS for identifying LPC are shown in Additional files 2, 3, 4, 5, 6, 7, 8, 9 : Videos S1–S8. The ROC curves for different datasets of LPAIDS, W, and N are shown in Fig. 3. The heat maps generated from LPAIDS are shown in Fig. 4.

**Table 2:** Performance comparison among LPAIDS, model W, and model N

	Accuracy (95% CI)	Sensitivity (95% CI)	Specificity (95% CI)	PPV (95% CI)	NPV (95% CI)	AUC
Images						
LPAIDS (All images)	0·956 (0·951–0·960)	0·948 (0·941–0·955)	0·964 (0·958–0·970)	0·965 (0·959–0·971)	0·946 (0·939–0·953)	0·974
Model W (WLI images)	0·948 (0·942–0·954)	0·885 (0·870–0·899)	0·988 (0·984–0·992)	0·978 (0·971–0·985)	0·932 (0·923–0·941)	0·955
LPAIDS (WLI images)	0·957 (0·951–0·962)	0·918 (0·906–0·931)	0·980 (0·975–0·985)	0·967 (0·959–0·975)	0·950 (0·943–0·958)	0·964
Model N (NBI images)	0·935 (0·926–0·945)	0·978 (0·971–0·984)	0·798 (0·765–0·831)	0·940 (0·930–0·951)	0·917 (0·893–0·941)	0·978
LPAIDS (NBI images)	0·954 (0·946–0·962)	0·977 (0·971–0·984)	0·878 (0·852–0·905)	0·963 (0·955–0·971)	0·923 (0·900–0·945)	0·981
Videos						
LPAIDS (All videos)	0·949 (0·931–0·968)	0·948 (0·922–0·974)	0·951 (0·924–0·977)	0·955 (0·930–0·979)	0·943 (0·916–0·971)	0·974
Model W (WLI videos)	0·945 (0·921–0·968)	0·886 (0·835–0·937)	0·986 (0·970–1·000)	0·978 (0·953–1·000)	0·925 (0·890–0·959)	0·948
LPAIDS (WLI videos)	0·950 (0.928–0.973)	0·919 (0·876–0·963)	0·972 (0·949–0·994)	0·958 (0·925–0·991)	0·945 (0·915–0·975)	0·968
Model N (NBI videos)	0·926 (0·889–0·963)	0·971 (0·943–0·999)	0·804 (0·695–0·913)	0·931 (0·890–0·972)	0·911 (0·828–0·994)	0·974
LPAIDS (NBI videos)	0·947 (0·916–0·979)	0·978 (0·954–1·000)	0·863 (0·768–0·957)	0·951 (0·916–0·986)	0·936 (0·866–1·000)	0·974

AUC: area under the curve; PPV, positive predictive value;CI: confidence interval; LPAIDS: Laryngopharyngeal Artificial Intelligence Diagnostic System; NBI: narrow-band imaging; NPV, negative predictive value; WLI: white light imaging

**Fig. 5:**
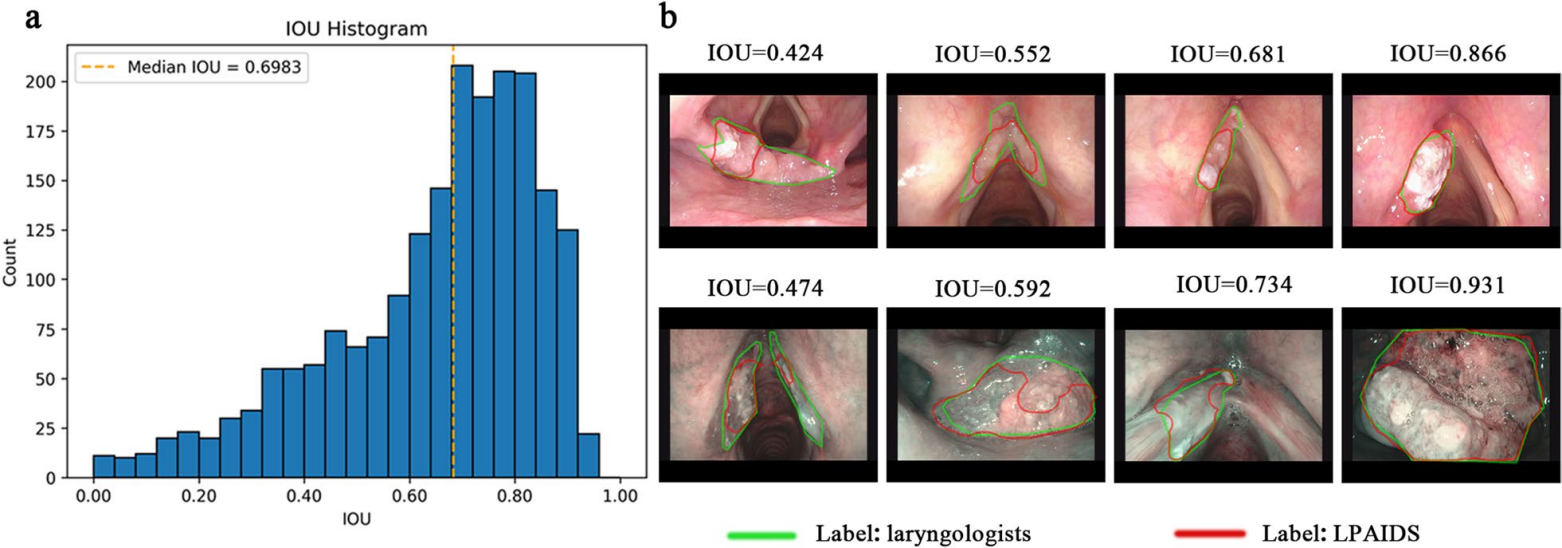
The accuracy of LPAIDS in segmentation of laryngopharyngeal cancer regions. **a** The distribution of IOU for the internal image test sets. **b** Representative prediction results correspond to various segmentation performances of LPAIDS for laryngopharyngeal cancer segmentation. The green line was labeled by the laryngoscopists, and the red line was labeled by LPAIDS automatic calculation. LPAIDS: Laryngopharyngeal Artificial Intelligence Diagnostic System; IOU: Intersection-Over-Union

We further evaluated the segmentation performance of LPAIDS in the positive pathological tissues. The LPAIDS-predicted segmented regions of LPC lesions were highly consistent with the areas labelled by laryngologists, with a median IOU of 0·698 in the internal temporal test sets (Fig. 5).

### Performance of the LPAIDS in external sets

**Fig. 6:**
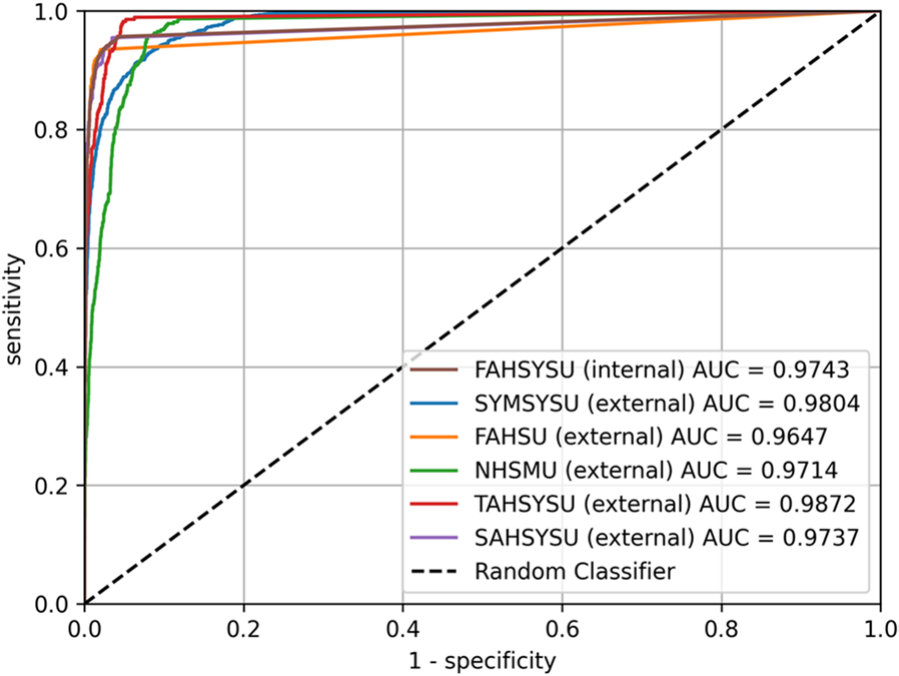
ROC curves illustrating the performance of LPAIDS for identifying laryngopharyngeal cancer in multicentre imaging datasets. FAHSU: First Affiliated Hospital of Shenzhen University; LPAIDS: FAHSYSU: First Affiliated Hospital of Sun Yat-sen University; Laryngopharyngeal Artificial Intelligence Diagnostic System; NHSMU: Nanfang Hospital of Southern Medical University; ROC: receiver operating characteristic; SAHSYSU: Sixth Affiliated Hospital of Sun Yat-sen University; SYMSYSU: Sun Yat-sen Memorial Hospital of Sun Yat-sen University; TAHSYSU: Third Affiliated Hospital of Sun Yat-sen University

In the external test sets, LPAIDS showed robust and generalisable performance in identifying patients with LPC (Table 3). The diagnostic accuracy was 0·949 (95% CI 0·944–0·954), 0·951 (95% CI 0·946–0·956), 0·984 (95% CI 0·981–0·988), 0.980 (95% CI 0·975–0·985), and 0·976 (95% CI 0·970–0·982) for the Sun Yat-sen Memorial Hospital, NHSMU, FAHSU, Third Affiliated Hospital, and Sixth Affiliated Hospital, respectively. All external datasets’ sensitivity, specificity, and NPV were higher than 0·90. The PPV varied across the datasets from 0·538 (95% CI 0·500–0·577) in the NHSMU to 0·898 (95% CI 0·865–0·930) in the SAHSYSU. Nevertheless, the proportion of false-positive cases was < 10% in all external test sets (Additional file 1: Figure s1). The most common cause of false positives were leucoplakia and normal anatomy (e.g., aryepiglottic folds and pyriform sinus) affected by light or secretions (Additional file 1: Figure S2). Furthermore, the high AUC values (0·965–0·987) also indicated the excellent diagnostic performance of LPAIDS in the five external test sets (Fig. 6).

**Table 3:** Performance of LPAIDS in different validation datasets

	FAHSYSU validation	External validation				
	Internal validation	SYMSYSU	NHSMU	FAHSU	TAHSYSU	SAHSYSU
Accuracy (95% CI)	0·956 (0·951–0·960)	0·949 (0·944–0·954)	0·951 (0·946–0·956)	0·984 (0·981–0·988)	0·980 (0·975–0·985)	0·976 (0·970–0·982)
Sensitivity (95% CI)	0·948 (0·941–0·955)	0·961 (0·950–0·971)	0·920 (0·893–0·948)	0·927 (0·896–0·959)	0·986 (0·972–1·000)	0·901 (0·868–0·933)
Specificity (95% CI)	0·964 (0·958–0·970)	0·946 (0·941–0·952)	0·953 (0·948–0·958)	0·987 (0·984–0·990)	0·979 (0·974–0·984)	0·986 (0·982–0·991)
PPV (95% CI)	0·965 (0·959–0·971)	0·793 (0·773–0·813)	0·538 (0·500–0·577)	0·796 (0·751–0·841)	0·821 (0·780–0·862)	0·898 (0·865–0·930)
NPV (95% CI)	0·946 (0·939–0·953)	0·991 (0·989–0·994)	0·995 (0·993–0·997)	0·996 (0·994–0·998)	0·999 (0·997–1·000)	0·987 (0·982–0·991)
AUC	0·974	0·980	0·965	0·971	0·987	0·974

AUC, area under the curve; CI: confidence interval; FAHSU: First Affiliated Hospital of Shenzhen University; FAHSYSU: First Affiliated Hospital of Sun Yat-sen University; LPAIDS: Laryngopharyngeal Artificial Intelligence Diagnostic System; NHSMU: Nanfang Hospital of Southern Medical University; NPV, negative predictive value; PPV, positive predictive value; SAHSYSU: Sixth Affiliated Hospital of Sun Yat-sen University; SYMSYSU: Sun Yat-sen Memorial Hospital of Sun Yat-sen University; TAHSYSU: Third Affiliated Hospital of Sun Yat-sen University

### Comparison between the LPAIDS and laryngologists in videos

**Fig. 7:**
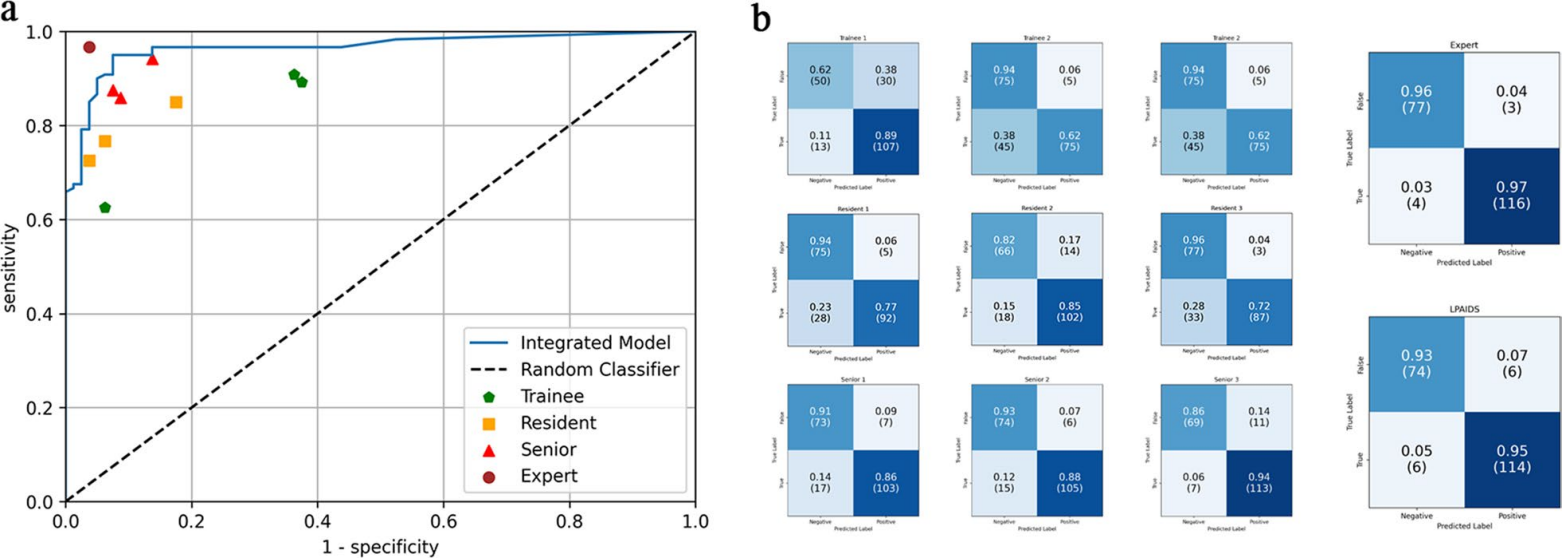
Diagnostic performance for identifying laryngopharyngeal cancer between the LPAIDS and laryngologists in 200 videos. **a** Receiver operating characteristic curves of LPAIDS, expert, senior, laryngologist residents, and trainees for comparison of the diagnostic performance. **b** Confusion matrices obtained by LPAIDS and ten laryngologists with varying degrees of expertise. Expert: a professor with > 20 years of experience in endoscopic procedures. Senior: attending doctors with more than five years of experience who had completed clinical and specific endoscopic training. Residents: residents with more than three years of endoscopic experience. Trainee: internsone year of endoscopic experience. LPAIDS: Laryngopharyngeal Artificial Intelligence Diagnostic System

To further verify the diagnostic validity of LPAIDS, we used 200 de-identified videos from the temporal test sets to compare the diagnostic performances of LPAIDS and laryngologists. The 200 videos comprised 120 LPC (including 66 WLI and 54 NBI videos), 55 benign disease (including 31 WLI and 24 NBI videos), and 25 normal (including 18 WLI and seven NBI videos) videos. The LPAIDS and laryngologists’ test results for identifying LPC are shown in Table 4. LPAIDS correctly diagnosed LPC with accuracy, sensitivity, specificity, PPV, and NPV of 0·940 (95% CI 0·907–0·973), 0·950 (95% CI 0·911–0·989), 0·925 (95% CI 0·867–0·983), 0·950 (95% CI 0·911–0·989), and 0·925 (95% CI 0·867–0·983), respectively. For comparison, as shown in Fig. 7a and Table 4, the diagnostic accuracy of LPAIDS was comparable to that of experts (0·965 [95% CI 0·940–0·990], *p* = 0·063) but significantly better than that of senior laryngologists (0·895 [95% CI 0·870–0·920], *p* = 0·001), laryngologist residents (0·832 [95% CI 0·802–0·862], *p* < 0·0001), and trainees (0·778 [95% CI 0·745–0·812], *p* < 0·0001). Details of the diagnostic performance of these 10 laryngologists are shown in Fig. 7b and Additional file 1: Table S1.

**Table 4:** Comparison between LPAIDS and laryngologists in 200 videos

	Accuracy (95% CI)	Sensitivity (95% CI)	Specificity (95% CI)	PPV (95% CI)	NPV (95% CI)
LPAIDS	0·940 (0·907–0·973)	0·950 (0·911–0·989)	0·925 (0·867–0·983)	0·950 (0·911–0·989)	0·925 (0·867–0·983)
Expert	0·965 (0·940–0·990)	0·967 (0·935–0·999)	0·963 (0·921–1·000)	0·975 (0·947–1·000)	0·951 (0·903–0·998)
Senior	0·895 (0·870–0·920)	0·892 (0·860–0·924)	0·900 (0·862–0·938)	0·930 (0·904–0·957)	0·847 (0·803–0·891)
Resident	0·832 (0·802–0·862)	0·781 (0·738–0·823)	0·908 (0·872–0·945)	0·927 (0·898–0·957)	0·734 (0·684–0·784)
Trainee	0·778 (0·745–0·812)	0.808 (0·768–0·849)	0·733 (0·677–0·789)	0·820 (0·780–0·860)	0·718 (0·662–0·775)

CI: confidence interval; LPAIDS: Laryngopharyngeal Artificial Intelligence Diagnostic System; NPV, negative predictive value; PPV, positive predictive value

Furthermore, we calculated the intra-observer agreement between the LPAIDS and laryngologists (Additional file 1: Table S2). The results showed that expert laryngologists achieved significantly higher diagnostic consistency (k = 0 ·948) than senior laryngologists (k: 0·755–0·811), laryngologist residents (k: 0·667–0·711), and trainees (k: 0·514–0·610). Additionally, the inter-observer agreement was higher for senior laryngologists (k: 0·687–0·775) than for laryngologist residents (k: 0·626–0·769) and trainees (k:0·349–0·453).

## Discussion

In this study, we developed a DCNN-based intelligent diagnosis system for LPC called LPAIDS, which incorporated both WLI and NBI images to automatically identify patients with LPC and was trained and validated across six hospitals. The system showed promising diagnostic performance in six independent test sets, with satisfactory accuracy (0·949–0·984), sensitivity (0·901–0·986), specificity (0·946–0·987), and AUC values (0·965–0·987). In a human–machine competition using an independent video test set, the diagnostic performance of LPAIDS was comparable to that of expert laryngologists and outperformed those of other laryngologists with different qualifications. To the best of our knowledge, this is the most extensive study in the field of AI guided for detecting LPC lesions based on laryngopharyngeal endoscopic images.

The screening and diagnosis of laryngopharyngeal carcinoma primarily rely on laryngoscopy and pathological biopsy of the suspicious cancer tissue under the guidance of laryngoscopy [29], and this subjective examination largely depends on the skills and experience of laryngologists, which increases the possibility of missed diagnosis and repeat unnecessary biopsy. The manifestation of early LPC is subtle mucosal changes under WLI, and combined with the application of NBI, it can enhance the visualisation of submucosal microvascular morphology; thick black spots can be observed within and surrounding malignant lesions [30, 31], which improves the detection rate of LPC. However, this technology suffers from a relatively long learning curve and is hampered by the need for expertise and intensive training for optical image interpretation [32]. In contrast, our system can recognise WLI and NBI images simultaneously with nearly no requirements for training and experience for laryngologists, achieving a high diagnostic accuracy similar to that of experts and better than that of non-experts in identifying LPC. This shows extraordinary potential for diagnosing LPC, particularly in developing countries or areas with an unbalanced distribution of medical resources. LPAIDS can help bridge the diagnostic gap between national and primary care hospitals and improve the diagnostic level of laryngologists lacking extensive experience and training.

Recently, in the field of endoscopy, the computer-aided diagnosis of gastrointestinal tumours has made remarkable progress [33–36]. Several preliminary studies have verified the feasibility of this method in the auxiliary diagnosis of LCA. Ren et al. established a CNN-based classifier to classify laryngeal disease [23]. Furthermore, Cho et al. applied a deep learning model to discriminate various laryngeal diseases except for malignancy [37]. They all reported high accuracy rates. However, in these two retrospective single-institutional studies, the validation set was a small subset random self of all images in the collection. This suggests that several images of one patient were distributed across both the training and validation sets, leading to an overestimation of the test results. The training and testing of our model adopted time-series sets, and all training, validation, and testing images were collected at different periods, which were completely independent and could simulate the datasets in prospective clinical trials with more objective and convincing results. Xiong et al. developed a model based on a DCNN using WLI images to diagnose LCA with an accuracy of 0·867 [25]. Additionally, He et al. developed a CNN model using NBI scans to identify patients with LCA, with an AUC of 0·873 in an independent test set [38]. Their studies were based on the diagnosis of a single imaging mode, which may lead to the omission of the focal features of the lesion, weakening the performance of AI-assisted diagnosis. Furthermore, both studies were only applicable to the detection of still images, which limits their practicality in clinical applications. The clinical application of AI requires the ability to analyse and diagnose complex situations in real time. The video contains multiple angles of the lesion and more complex diagnostic settings closer to the actual clinical environment. A pilot study by Azam et al. used 624 video frames of LCA to develop a YOLO ensemble model to attempt the automatic detection of LCA in real time [24]. This study focused on the automatic segmentation of tumour lesions using only LCA video frames, achieving an accuracy of 0·66 in 57 testing images, and verified the real-time processing performance of the model on six video laryngoscopes. Due to the small sample size and lack of controls, these results and their feasibility in clinical application for auxiliary diagnosis of LCA should be treated cautiously. The system we developed analysed one video frame that required only 26 ms, with an average of 38 video frames that can be identified per second, achieving the performance requirements required for real-time detection. Furthermore, our approach achieved a diagnostic accuracy of 0·949 in an independent video test set with 551 videos, demonstrating real-time dynamic recognition ability. Therefore, our system is more reliable for diagnosing LPC in real time and has a higher clinical utility than previously reported models.

Our system achieved satisfactory diagnostic performance with high accuracy on both image-test sets (0·956 [95% CI 0·951–0·960]) and video-test sets (0·949 [95% CI 0·931–0·968]), which depended on the subsequent improvement to the U-Net. We extracted two features from WLI and NBI images, respectively, which independently represented different data types, and further fused the two features. Compared with the models simply using mixed images, the LPAIDS led to more accurate predictions either in WLI or NBI images. Furthermore, integrating the two features is based on linear layers, which uses less time than feature extraction from multimodal data. The fast integration ensures that the LPAIDS can meet demanding requirements in real time. The stability and robustness of the model were validated using five other independent external validation sets. Moreover, the diagnostic performance of our system was comparable to that of experts and higher than that of non-experts. We used the Cohen kappa coefficient to assess the stability between the system and the laryngologists. We found that the expert achieved significant intra-observer consistency (k = 0·948), which was higher than that of senior laryngologists (k: 0·755–0·811), laryngologist residents (k: 0·667–0·711), and trainees (k: 0·514–0·610).

Despite these promising results, some limitations remain. First, this was a retrospective study, which may have a certain degree of selection bias, and the excellent performance of the LPAIDS cannot entirely reflect actual clinical application. Time-series sets were used to avoid such problems in the study design. Additionally, we designed and prepared a multicentre prospective randomised controlled trial to verify the applicability of this system in a clinical setting. Second, our dataset mostly comprises high-quality laryngoscopy images, which may limit the scope of use of this system. However, our test set used images acquired by different endoscopy systems from various institutions, such as Olympus and Xion, which account for most of the endoscopy market. We will collect more images of varying quality to enhance the generalisation ability of our system. Third, although we used a video test to demonstrate the real-time detection performance of the system, the clipped video only contained lesions, and the real-time application ability in actual clinical practice should be evaluated. We will further work on embedding the system into the endoscopic system to output prediction results while performing laryngoscopy and evaluating the model’s reliability.

## Conclusion

We developed a DCNN-based system for the real-time detection of LPCs. The system could recognise WLI and NBI imaging modalities simultaneously, achieving high accuracy and sensitivity in independent image and video test sets. The diagnostic efficiency was equivalent to that of experts and better than non-experts. However, this study still needs multicentre prospective verification to provide high-level evidence for detecting LPC in actual clinical practice. We believe that LPAIDS has excellent potential for aiding the diagnosis of LPC and reducing the burden on laryngologists.

### Supplementary Information

**Additional file 1: Figure S1.** Confusion matrixes of LPAIDS in identifying laryngopharyngeal cancer in multicentre imaging datasets. **Figure S2.** Common false positive cases. **Table S1.** Performance of the LPAIDS versus laryngologists in 200 videos. **Table S2.** Inter-observer and intra-observer agreement of the LPAIDS and laryngologists in the human-machine competition dataset

**Additional file 2: Video S1.** Laryngeal cancer (glottic carcinoma) detection of LPAIDS in the WLI video

**Additional file 3: Video S2.** Laryngeal cancer (glottic carcinoma) detection of LPAIDS in the NBI video

**Additional file 4: Video S3.** Laryngeal cancer (glottic carcinoma) detection of LPAIDS in the WLI video

**Additional file 5: Video S4.** Laryngeal cancer (glottic carcinoma) detection of LPAIDS in the NBI video

**Additional file 6: Video S5.** Laryngeal cancer (supraglottic carcinoma) detection of LPAIDS in the WLI video

**Additional file 7: Video S6.** Laryngeal cancer (supraglottic carcinoma) detection of LPAIDS in the NBI video

**Additional file 8: Video S7.** Hypopharyngeal cancer detection of LPAIDS in the WLI video

**Additional file 9: Video S8.** Hypopharyngeal cancer detection of LPAIDS in the NBI video

## Data Availability

Due to patient privacy, all the datasets generated or analyzed in this study are available from the corresponding author upon reasonable request by The First Affiliated Hospital of Sun Yat-sen University (leiwb@mail.sysu.edu.cn).

## Abbreviations

AI: Artificial intelligence
AUC: Area under the ROC curve
CI: Confidence interval
DCNN: Deep convolutional neural networks
FAHSU: First Affiliated Hospital of Shenzhen University;
FAHSYSU: First Affiliated Hospital of Sun Yat-sen University;
IOU: Intersection-over-union
LCA: Laryngeal cancer
LPAIDS: Laryngopharyngeal Artificial Intelligence Diagnostic System
LPC: Laryngopharyngeal cancer
NBI: Narrow-band imaging
NHSMU: Nanfang Hospital of Southern Medical University;
NPV: Negative predictive value
PPV: Positive predictive value
ReLU: Rectified linear unit
ROC: Receiver operating characteristic
SAHSYSU: Sixth Affiliated Hospital of Sun Yat-sen University
SYMSYSU: Sun Yat-sen Memorial Hospital of Sun Yat-sen University;
TAHSYSU: Third Affiliated Hospital of Sun Yat-sen University;
WLI: White light imaging

## References

[1] Sung H, Ferlay J, Siegel RL (2021). Global cancer statistics 2020: GLOBOCAN estimates of incidence and mortality worldwide for 36 cancers in 185 countries. CA Cancer J Clin.

[2] Steuer CE, El-Deiry M, Parks JR, Higgins KA, Saba NF (2017). An update on larynx cancer. CA Cancer J Clin.

[3] Marioni G, Marchese-Ragona R, Cartei G (2006). Current opinion in diagnosis and treatment of laryngeal carcinoma. Cancer Treat Rev.

[4] Mannelli G, Cecconi L, Gallo O (2016). Laryngeal preneoplastic lesions and cancer: challenging diagnosis. Qualitative literature review and meta-analysis. Crit Rev Oncol Hematol..

[5] Krausert CR, Olszewski AE, Taylor LN (2011). Mucosal wave measurement and visualization techniques. J Voice.

[6] Ni XG, Zhang QQ, Wang GQ (2016). Narrow band imaging versus autofluorescence imaging for head and neck squamous cell carcinoma detection: a prospective study. J Laryngol Otol.

[7] Zwakenberg MA, Halmos GB, Wedman J (2021). Evaluating laryngopharyngeal tumor extension using narrow band imaging versus conventional white light imaging. Laryngoscope.

[8] Brennan MT, Treister NS, Sollecito TP (2021). Epidemiologic factors in patients with advanced head and neck cancer treated with radiation therapy. Head Neck.

[9] Driessen CML, Leijendeckers J, Snik AD (2019). Ototoxicity in locally advanced head and neck cancer patients treated with induction chemotherapy followed by intermediate or high-dose cisplatin-based chemoradiotherapy. Head Neck..

[10] Marur S, Forastiere AA (2016). Head and neck squamous cell carcinoma: update on epidemiology, diagnosis, and treatment. Mayo Clin Proc.

[11] Gugatschka M, Kiesler K, Beham A (2008). Hyperplastic epithelial lesions of the vocal folds: combined use of exfoliative cytology and laryngostroboscopy in differential diagnosis. Eur Arch Otorhinolaryngol.

[12] Cosway B, Drinnan M, Paleri V (2016). Narrow band imaging for the diagnosis of head and neck squamous cell carcinoma: a systematic review. Head Neck.

[13] Kim DH, Kim Y, Kim SW, Hwang SH (2020). Use of narrowband imaging for the diagnosis and screening of laryngeal cancer: a systematic review and meta-analysis. Head Neck.

[14] Ni XG, Wang GQ (2016). The role of narrow band imaging in head and neck cancers. Curr Oncol Rep.

[15] Chen J, Li Z, Wu T, Chen X (2023). Accuracy of narrow-band imaging for diagnosing malignant transformation of vocal cord leukoplakia: a systematic review and meta-analysis. Laryngoscope Investig Otolaryngol.

[16] Ni XG, Wang GQ, Hu FY (2019). Clinical utility and effectiveness of a training programme in the application of a new classification of narrow-band imaging for vocal cord leukoplakia: a multicentre study. Clin Otolaryngol.

[17] Liang H, Tsui BY, Ni H (2019). Evaluation and accurate diagnoses of pediatric diseases using artificial intelligence. Nat Med.

[18] Mathenge WC (2019). Artificial intelligence for diabetic retinopathy screening in Africa. Lancet Digit Health.

[19] Dey D, Slomka PJ, Leeson P (2019). Artificial intelligence in cardiovascular imaging: JACC State-of-the-Art review. J Am Coll Cardiol.

[20] Zhang B, Jin Z, Zhang S (2021). A deep-learning model to assist thyroid nodule diagnosis and management. Lancet Digit Health.

[21] Foersch S, Eckstein M, Wagner DC (2021). Deep learning for diagnosis and survival prediction in soft tissue sarcoma. Ann Oncol.

[22] Luo H, Xu G, Li C (2019). Real-time artificial intelligence for detection of upper gastrointestinal cancer by endoscopy: a multicentre, case-control, diagnostic study. Lancet Oncol.

[23] Ren J, Jing X, Wang J (2020). Automatic recognition of laryngoscopic images using a deep-learning technique. Laryngoscope.

[24] Azam MA, Sampieri C, Ioppi A (2022). Deep learning applied to white light and narrow band imaging videolaryngoscopy: toward real-time laryngeal cancer detection. Laryngoscope.

[25] Xiong H, Lin P, Yu JG (2019). Computer-aided diagnosis of laryngeal cancer via deep learning based on laryngoscopic images. EBioMedicine.

[26] Kwon I, Wang SG, Shin SC (2022). Diagnosis of early glottic cancer using laryngeal image and voice based on ensemble learning of convolutional neural network classifiers. J Voice.

[27] Inaba A, Hori K, Yoda Y (2020). Artificial intelligence system for detecting superficial laryngopharyngeal cancer with high efficiency of deep learning. Head Neck.

[28] Ronneberger O, Fischer P, Brox T. U-Net. Convolutional Networks for Biomedical Image Segmentation. International Conference on Medical Image Computing and Computer-Assisted Intervention. 2015; 2015.

[29] Stachler RJ, Francis DO, Schwartz SR (2018). Clinical practice guideline: hoarseness (Dysphonia) (Update). Otolaryngol Head Neck Surg..

[30] Zwakenberg MA, Dikkers FG, Wedman J, Halmos GB, van der Laan BF, Plaat BE (2016). Narrow band imaging improves observer reliability in evaluation of upper aerodigestive tract lesions. Laryngoscope.

[31] Vilaseca I, Valls-Mateus M, Nogués A (2017). Usefulness of office examination with narrow band imaging for the diagnosis of head and neck squamous cell carcinoma and follow-up of premalignant lesions. Head Neck.

[32] Irjala H, Matar N, Remacle M, Georges L (2011). Pharyngo-laryngeal examination with the narrow band imaging technology: early experience. Eur Arch Otorhinolaryngol.

[33] He X, Wu L, Dong Z (2022). Real-time use of artificial intelligence for diagnosing early gastric cancer by magnifying image-enhanced endoscopy: a multicenter diagnostic study (with videos). Gastrointest Endosc.

[34] Lu Z, Xu Y, Yao L (2022). Real-time automated diagnosis of colorectal cancer invasion depth using a deep learning model with multimodal data (with video). Gastrointest Endosc.

[35] Xu M, Zhou W, Wu L (2021). Artificial intelligence in the diagnosis of gastric precancerous conditions by image-enhanced endoscopy: a multicenter, diagnostic study (with video). Gastrointest Endosc.

[36] Tang D, Wang L, Ling T (2020). Development and validation of a real-time artificial intelligence-assisted system for detecting early gastric cancer: a multicentre retrospective diagnostic study. EBioMedicine.

[37] Cho WK, Lee YJ, Joo HA (2021). Diagnostic accuracies of laryngeal diseases using a convolutional neural network-based image classification system. Laryngoscope.

[38] He Y, Cheng Y, Huang Z (2021). A deep convolutional neural network-based method for laryngeal squamous cell carcinoma diagnosis. Ann Transl Med.

