# Supplementary material for: Real-time detection of laryngopharyngeal cancer using an artificial intelligence-assisted system with multimodal data
Source: J Transl Med. 2023 Oct 7;21:698. doi: 10.1186/s12967-023-04572-y (PMC10559609; doi:10.1186/s12967-023-04572-y)
Supplement: Supplementary file 1 — Additional file 1: Figure S1. Confusion matrixes of LPAIDS in identifying laryngopharyngeal cancer in multicentre imaging datasets. Figure S2. Common false positive cases. Table S1. Performance of the LPAIDS versus laryngologists in 200 videos. Table S2. Inter-observer and intra-observer agreement of the LPAIDS and laryngologists in the human-machine competition dataset [file 12967_2023_4572_MOESM1_ESM.docx]

**Supplementary materials**

**Figure S1**

**
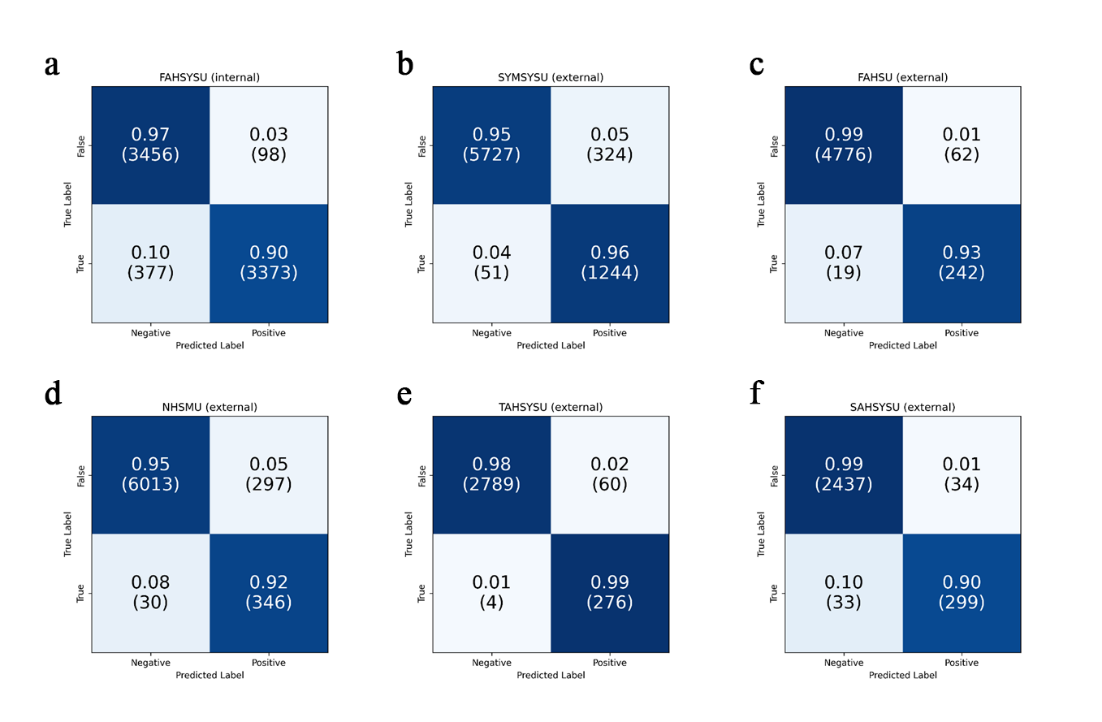
**

**Figure S1. Confusion matrixes of LPAIDS in identifying laryngopharyngeal cancer in multicentre imaging datasets.**

(a) The confusion matrix of LPAIDS in FAHSYSU temporal validation dataset. (b) The confusion matrix of LPAIDS in SYMSYSU external validation dataset. (c) The confusion matrix of LPAIDS in FAHSU external validation dataset. (d) The confusion matrix of LPAIDS in NHSMU external validation dataset. (e) The confusion matrix of LPAIDS in TAHSYSU external validation dataset. (f) The confusion matrix of LPAIDS in SAHSYSU external validation dataset. LPAIDS: Laryngopharyngeal Artificial Intelligence Diagnostic System; FAHSYSU: First Affiliated Hospital of Sun Yat-sen University; SYMSYSU: Sun Yat-sen Memorial Hospital of Sun Yat-sen University; NHSMU: Nanfang Hospital of Southern Medical University; FAHSU: First Affiliated Hospital of Shenzhen University; TAHSYSU: Third Affiliated Hospital of Sun Yat-sen University; SAHSYSU: Sixth Affiliated Hospital of Sun Yat-sen University.


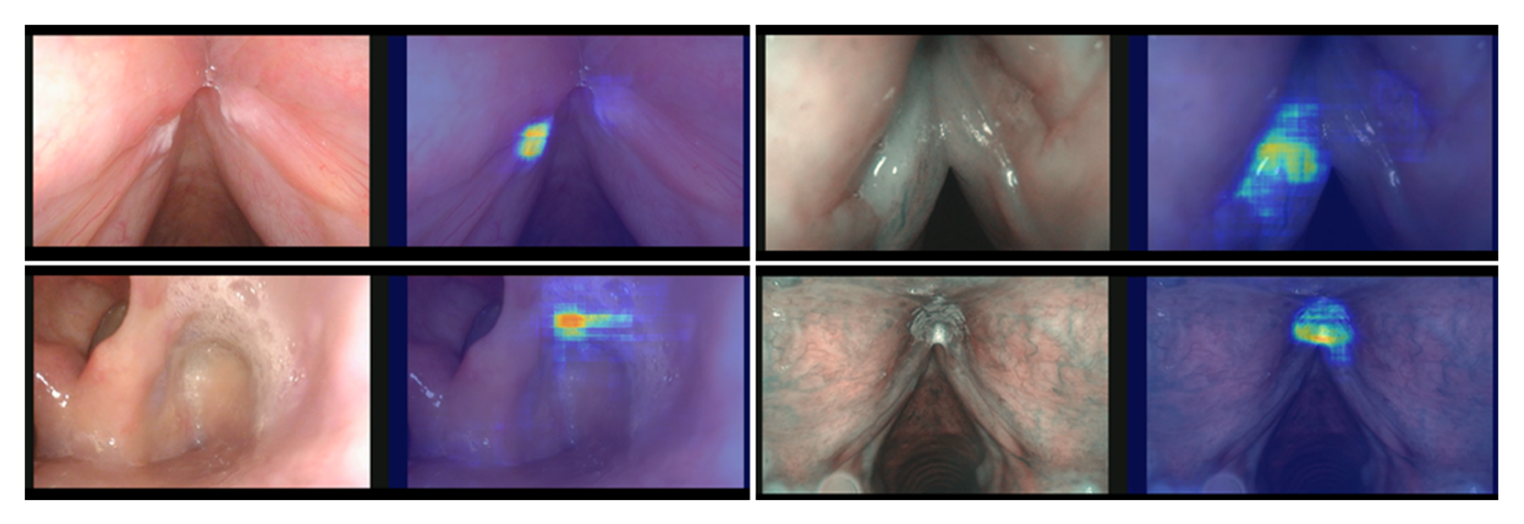


**Figure S2. Common false positive cases.**

**Video Legends**

**Video 1: Laryngeal cancer (glottic carcinoma) detection of LPAIDS in the WLI video.**

LPAIDS: Laryngopharyngeal Artificial Intelligence Diagnostic System.

**Video 2: Laryngeal cancer (glottic carcinoma) detection of LPAIDS in the NBI video.**

LPAIDS: Laryngopharyngeal Artificial Intelligence Diagnostic System.

**Video 3: Laryngeal cancer (glottic carcinoma) detection of LPAIDS in the WLI video.**

LPAIDS: Laryngopharyngeal Artificial Intelligence Diagnostic System.

**Video 4: Laryngeal cancer (glottic carcinoma) detection of LPAIDS in the NBI video.**

LPAIDS: Laryngopharyngeal Artificial Intelligence Diagnostic System.

**Video 5: Laryngeal cancer (supraglottic carcinoma) detection of LPAIDS in the WLI video.**

LPAIDS: Laryngopharyngeal Artificial Intelligence Diagnostic System.

**Video 6: Laryngeal cancer (supraglottic carcinoma) detection of LPAIDS in the NBI video.**

LPAIDS: Laryngopharyngeal Artificial Intelligence Diagnostic System.

**Video 7: Hypopharyngeal cancer detection of LPAIDS in the WLI video.**

LPAIDS: Laryngopharyngeal Artificial Intelligence Diagnostic System.

**Video 8: Hypopharyngeal cancer detection of LPAIDS in the NBI video.**

LPAIDS: Laryngopharyngeal Artificial Intelligence Diagnostic System.

**Table S1 Performance of the LPAIDS versus laryngologists in 200 videos**

|  | **Accuracy (95% CI)** | **Sensitivity (95% CI)** | **Specificity (95% CI)** | **PPV (95% CI)** | **NPV (95% CI)** |
| --- | --- | --- | --- | --- | --- |
| LPAIDS | 0.940 (0.907-0.973) | 0.950 (0.911-0.989) | 0.925 (0.867-0.983) | 0.950 (0.911-0.989) | 0.925 (0.867-0.983) |
| Expert | 0.965 (0.940-0.990) | 0.967 (0.935-0.999) | 0.963 (0.921-1.000) | 0.975 (0.947-1.000) | 0.951 (0.903-0.998) |
| Senior |  |  |  |  |  |
| Senior 1 | 0.880 (0.835-0.925) | 0.858 (0.796-0.921) | 0.912 (0.851-0.974) | 0.936 (0.891-0.982) | 0.811 (0.730-0.892) |
| Senior 2 | 0.895 (0.853-0.937) | 0.875 (0.816-0.934) | 0.925 (0.867-0.983) | 0.946 (0.904-0.988) | 0.831 (0.754-0.909) |
| Senior 3 | 0.910 (0.870-0.950) | 0.942 (0.900-0.984) | 0.863 (0.787-0.938) | 0.911 (0.861-0.961) | 0.908 (0.843-0.973) |
| Resident |  |  |  |  |  |
| Resident 1 | 0.835 (0.784-0.886) | 0.767 (0.691-0.842) | 0.938 (0.884-0.991) | 0.948 (0.904-0.992) | 0.728 (0.642-0.814) |
| Resident 2 | 0.840 (0.789-0.891) | 0.850 (0.786-0.914) | 0.825 (0.742-0.908) | 0.879 (0.820-0.939) | 0.786 (0.698-0.873) |
| Resident 3 | 0.820 (0.767-0.873) | 0.725 (0.645-0.805) | 0.963 (0.921-1.000) | 0.967 (0.930-1.000) | 0.700 (0.614-0.786) |
| Trainee |  |  |  |  |  |
| Trainee 1 | 0.785 (0.728-0.842) | 0.892 (0.836-0.947) | 0.625 (0.519-0.731) | 0.781 (0.712-0.850) | 0.794 (0.694-0.894) |
| Trainee 2 | 0.750 (0.690-0.810) | 0.625 (0.538-0.712) | 0.938 (0.884-0.991) | 0.938 (0.884-0.991) | 0.625 (0.538-0.712) |
| Trainee 3 | 0.800 (0.745-0.855) | 0.908 (0.857-0.960) | 0.637 (0.532-0.743) | 0.790 (0.722-0.858) | 0.823 (0.727-0.918) |

LPAIDS: Laryngopharyngeal Artificial Intelligence Diagnostic System; CI: confidence interval; PPV, positive predictive value; NPV, negative predictive value

**Table S2 Inter-observer and intra-observer agreement of the LPAIDS and laryngologists in the human-machine competition dataset**

|  | **Kappa value** | | | | | | | | | | | | | | | |
| --- | --- | --- | --- | --- | --- | --- | --- | --- | --- | --- | --- | --- | --- | --- | --- | --- |
|  | LPAIDS |  | Expert |  | Senior | | |  | Resident | | |  | Trainee | | |  |
|  |  |  |  |  | 1 | 2 | 3 |  | 1 | 2 | 3 |  | 6 | 7 | 8 |  |
| LPAIDS | / |  | / |  | / | / | / |  | / | / | / |  | / | / | / |  |
| Expert | 0.948 |  | / |  | / | / | / |  | / | / | / |  | / | / | / |  |
| Senior | | | | | | | | | | | | | | | | |
| 1 | 0.755 |  | / |  | / | / | / |  | / | / | / |  | / | / | / |  |
| 2 | 0.806 |  | / |  | 0.687 | / | / |  | / | / | / |  | / | / | / |  |
| 3 | 0.811 |  | / |  | 0.775 | 0.764 | / |  | / | / | / |  | / | / | / |  |
| Resident | | | | | | | | | | | | | | | | |
| 1 | 0.692 |  | / |  | / | / | / |  | / | / | / |  | / | / | / |  |
| 2 | 0.711 |  | / |  | / | / | / |  | 0.632 | / | / |  | / | / | / |  |
| 3 | 0.667 |  | / |  | / | / | / |  | 0.769 | 0.626 | / |  | / | / | / |  |
| Trainee | | | | | | | | | | | | | | | | |
| 1 | 0.514 |  | / | / | / | / | / |  | / | / | / |  | / | / | / |  |
| 2 | 0.519 |  | / | / | / | / | / |  | / | / | / |  | 0.358 | / | / |  |
| 3 | 0.610 |  | / | / | / | / | / |  | / | / | / |  | 0.453 | 0.349 | / |  |

LPAIDS: Laryngopharyngeal Artificial Intelligence Diagnostic System.
